# Frequent emergency department use by older adults with ambulatory care sensitive conditions: A population‐based cohort study

**DOI:** 10.1111/ggi.13875

**Published:** 2020-02-03

**Authors:** Isabelle Dufour, Yohann Chiu, Josiane Courteau, Maud‐Christine Chouinard, Nicole Dubuc, Catherine Hudon

**Affiliations:** ^1^ School of Nursing, Department of Health Sciences, Université de Sherbrooke, Sherbrooke Quebec Canada; ^2^ Department of Family and Emergency Medicine, Université de Sherbrooke, Sherbrooke Quebec Canada; ^3^ Department of Health Sciences, Université du Québec à Chicoutimi, Saguenay Quebec Canada; ^4^ PRIMUS Research Group, Centre de recherche du Centre hospitalier universitaire de Sherbrooke (CRCHUS)

**Keywords:** administrative database, aged, ambulatory care sensitive conditions, emergency, frequent use

## Abstract

**Aim:**

To identify factors associated with frequent emergency department (ED) use among older adults with ambulatory care sensitive conditions.

**Methods:**

This was a retrospective cohort study using databases from the Régie de l'assurance maladie du Québec. We included community‐dwelling individuals aged ≥65 years in the Province of Quebec (Canada), who consulted in ED at least once between 2012 and 2013 (index period), and were diagnosed with at least one ambulatory care sensitive condition in the 2 years preceding and including the index date (*n* = 264 473). We used a multivariate logistic regression model to evaluate the association between independent variables and being a frequent geriatric ED user, defined as four or more visits during the year after the index date.

**Results:**

Out of the total study population, 17 332 (6.6%) individuals were considered frequent ED users in the year after the index date, accounting for 38% of ED uses for this period. The main variables associated with frequent geriatric ED use were older age, presence of chronic obstructive pulmonary disorder or diabetes, higher comorbidity index, common mental health disorders, polypharmacy, higher number of past ED and specialist visits, rural residence, and higher material and social deprivation. Dementia was inversely associated with frequent ED use.

**Conclusions:**

Frequent geriatric ED users constitute a complex population whose characteristics need to be managed thoroughly in order to enhance the quality and efficiency of their care. Further studies should address their description in administrative databases so as to combine self‐perceived and professionally evaluated variables. **Geriatr Gerontol Int 2020; 20: 317–323**.

## Introduction

Approximately 6% of older adults are considered frequent users of emergency departments (ED), accounting for up to 30% of such use.^1^ Frequent geriatric users are defined as patients aged >65 years with numerous ED visits within a year‐long period, the most accepted definition being four or more visits.^1^ ED visits put older adults at risk of adverse effects, including hospitalization, frequent ED episodes, functional decline, and complications regarding treatments and procedures.^2^ The high use of ED by older adults also has implications for healthcare systems, some of which are already burdened with overcrowding.^3^ Although all ED visits by older adults are not preventable – related to conditions of higher severity – using these services does not always effectively fulfill the healthcare needs of these patients.^4^ This is particularly the case for the large proportion of frequent geriatric ED users diagnosed with ambulatory care sensitive conditions (ACSC). ACSC represent a range of chronic diseases considered to be optimally taken care of by timely and effective management in primary healthcare.^5^ It has been also documented that ACSC progression can result in complex multimorbidity problems, particularly among older adults. Adequate care in appropriate services can therefore prevent complications, as well as a certain proportion of hospitalizations and ED visits.^5^

A recent systematic review by Dufour *et al*. highlighted the main variables associated with frequent geriatric ED use, including a high number of past hospital and ED admissions, living in a rural area adjacent to an urban center, low income, and a high number of prescribed drugs.^6^ The authors also highlighted a knowledge gap in the description of frequent geriatric ED users, as variables, such as dementia and primary care use, received little attention.^6^

To bridge these gaps, we aimed to identify factors associated with frequent ED use among older adults with ACSC in the Province of Quebec, and to present their comprehensive portrait.

## Methods

### *Design and data source*

This was a retrospective cohort study, using data obtained by the provincial health information board (Régie de l'assurance maladie du Québec [RAMQ]), which provides universal health insurance to Quebec (Canada) residents (~8 000 000 inhabitants). The covered services are provided in a variety of settings, including ED, hospitals and medical clinics. The study is reported in accordance with the STROBE Statement (Strengthening the Reporting of Observational Studies in Epidemiology).

The RAMQ administrative health register gives access to a large range of variables including: (i) patient demographic information (date of birth and death, place of residence etc.); (ii) medical services register (data on any medical service provided by a fee‐for‐service physician in Quebec, including diagnosis coded according to the International Classification of Diseases 9 [ICD‐9]); (iii) provincial public drug insurance plan eligibility (insurance status etc.); (iv) pharmaceutical services (data on each drug claimed in a pharmacy); (v) MED‐ECHO registry (information on hospitalization, length of stay, main and up to 25 secondary diagnoses coded in ICD‐10); and (vi) APR‐DRG (All Patient Refined – Diagnosis Related Groups) offering additional information related to hospitalization, such as the severity of each episode. Patient data from these registers were linked using a unique encrypted identifier to provide information on demographic and medical characteristics.

### *Study population*

**Figure 1:**
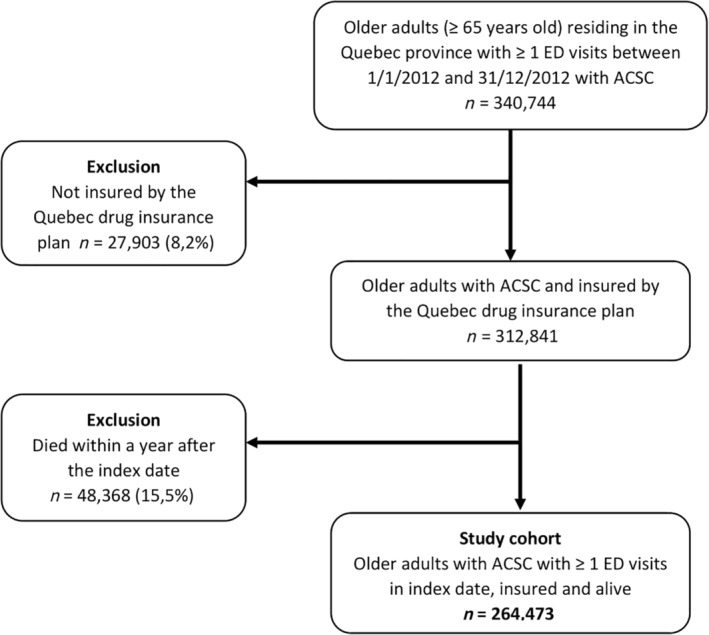
Flowchart of cohort selection. ACSC, ambulatory care sensitive conditions; ED, emergency department.

The study population included all community‐dwelling individuals age ≥65 years residing in the Province of Quebec (Canada) who consulted in an ED at least once between 1 January 2012 and 31 December 2013 (index period), and was diagnosed with at least one ACSC in the 2 years preceding and including the index date. The index date was defined as an ED visit, randomly chosen, during the index period. For the present study, we used the Canadian Institute for Health Information (2012) definition of ACSC.^7^ This includes coronary heart disease, congestive heart failure, chronic obstructive pulmonary disease (COPD), asthma, diabetes, high blood pressure and epilepsy. An individual was considered as having an ACSC if they met at least one of the following two criteria: (i) the presence of two diagnosis codes for the same ACSC entered in the medical services register on two different dates; and (ii) the presence of a primary or secondary diagnosis code for an ACSC during hospitalization (MED‐ECHO). Table 1 provides the list of ICD codes. We excluded patients not insured under the Quebec drug insurance plan and those who died within 365 days of the index date in order to reduce the risk of immortal bias (see Fig. 1 for selection details).

**Table 1:** International classification of diseases for ambulatory care sensitive conditions diagnoses used in the present study

	ICD‐9	ICD‐10
Coronary heart disease	410–414	I20–I25
Congestive heart failure	428, 518.4	I50, J81
Chronic obstructive pulmonary disease	490–492, 494, 496	J40–J44, J47
Asthma	493	J45
Diabetes	250	E10–E14
High blood pressure	401–405	I10–I13, I15
Epilepsy	345	G40–G41

ACSC, ambulatory care sensitive conditions; ICD‐9, International Classification of Diseases, 9th revision; ICD‐10, International Classification of Diseases, 10th revision.

### *Outcome and independent variables*

The main outcome of the present study is being a frequent geriatric ED user (dichotomic variable, yes/no). ED frequent use is defined as four or more visits during a 1‐year period.

The choice of independent variables was intended to yield a global portrait of the population. Age, sex, material and social deprivation quintiles (quintile 1 – least deprived – to quintile 5 –most deprived) and residential neighborhood characteristics (metropolitan area ≥100 000 inhabitants; small town: 10000–100 000 inhabitants; rural: <10 000 inhabitants) were all considered at the index date. ACSC type, mental and physical health diagnoses (e.g. dementia, chronic pain) were all identified in the 2 years preceding the index date (1 diagnosis during a hospitalization or a diagnosis mentioned at least twice in the medical services register). Past use of healthcare services (e.g. ED visits, hospitalization, consultations with specialists, general practitioner affiliation) was identified in the 2 years preceding the index date. Potentially inappropriate drug (e.g. opioids, benzodiazepines and antipsychotic drugs) and a number of other drugs (excluding topical, dermatological, ophthalmic and otic products) were calculated in the month preceding the index date. The number of drugs was divided into categories: polypharmacy (defined as the simultaneous use of 5–9 medications) and severe polypharmacy (defined as the simultaneous use of ≥10 medications).^8^ Finally, the comorbidity index score consisted of the Combined Comorbidity Index of Charlson and Elixhauser, as proposed by Simard *et al*. calculated in the 2 years preceding the index date and from which the ACSC were removed.^9^

### *Statistical analysis*

First, we described the individual's characteristics by ED‐user type: (i) infrequent users (<4 visits during the year after the index date); or (ii) frequent users (≥4 visits during the year after the index date). The difference between subgroups was tested using the χ^2^‐test for categorical variables, and the Kruskal–Wallis test for continuous variables.

Second, we used a multivariate regression model to test the association between the independent variables and being a frequent geriatric ED user. The final model used a backward selection method and reported odd ratios (OR) with the associated 99% confidence intervals (CI). As logistic regression models are sensitive to large sample sizes, we chose a significance level of 0.01 to decrease the risk of alpha error.^10^ The continuous independent variables not respecting the linearity assumption were categorized. Examination of the 2 × 2 matrix and the variance inflation factor showed no multicollinearity issues.

### *Ethical consideration*

The ethics approval for this study was obtained from the ethics review board of the Université de Sherbrooke and by the Commission d'accès à l'information of Quebec.

## Results

The study cohort was composed of 264 473 older individuals, of which 17 332 (6.6%) were considered frequent ED users in the year after the index date. They accounted for 38% of ED use for this period. A low proportion of missing values was reported in the database – <0.5% – with a maximum of 5.1% for the social and material deprivation variables.

Table 2 gives the characteristics of the study cohort. Unlike infrequent users, frequent users presented a higher proportion of coronary heart disease (20.6% *vs* 8.8%), COPD (34.1% *vs* 15.9%), mental health disorders (common: 26.6% *vs* 15.2%; severe: 11% *vs* 5.3%), dementia (13.3% *vs* 7.9%), alcohol abuse (4.3% *vs* 1.8%) and severe polypharmacy (48.9% *vs* 26.5%). Furthermore, frequent users presented a higher comorbidity score and the highest proportion of past use of healthcare services, including ED visits, hospitalizations and lengths of stay, and visits to general practitioners and specialists. The results show statistically significant differences between the infrequent and frequent user groups for all variables expect sex (*P*‐value = 0.70).

**Table 2:** Characteristics of the study cohort

Variables	Total population	Infrequent users	Frequent users	*P*‐ value†
Total	264 473 (100)	247 141 (100)	17 332 (100)	–
Age	76.5 ± 7.7	76.5 ± 7.7	77.7 ± 7.8	<0.0001‡
Sex				
Male	117 412 (44.4)	109 742 (44.4)	7670 (44.3)	0.07
Female	147 061 (55.6)	137 399 (55.6)	9662 (55.8)	–
ACSC Coronary heart disease	25 404 (9.6)	21 826 (8.8)	3578 (20.6)	<0.0001
ACSC Congestive heart failure	87 947 (33.3)	79 814 (32.3)	8133 (49.9)	<0.0001
ACSC COPD	45 124 (17.1)	39 220 (15.9)	5904 (34.1)	<0.0001
ACSC Asthma	15 300 (5.8)	13 698 (5.5)	1602 (9.2)	<0.0001
ACSC Diabetes	90 469 (34.2)	83 403 (33.8)	7066 (40.8)	<0.0001
ACSC High blood pressure	169 141 (64.0)	157 009 (63.5)	12 132 (70.0)	<0.0001
ACSC Epilepsy	3827 (1.5)	3382 (1.4)	445 (2.6)	<0.0001
Comorbidity index				
0	92 259 (34.9)	89 476 (36.2)	2783 (16.1)	<0.0001
1–2	72 875 (27.6)	69 069 (28.0)	3806 (22.0)	–
3–4	40 904 (15.5)	37 565 (15.2)	3339 (19.3)	–
≥5	58 435 (22.1)	51 031 (20.7)	7404 (42.7)	–
Common mental‐health disorder	42 170 (15.9)	37 554 (15.2)	4616 (26.6)	<0.0001
Severe mental disorder	15 114 (5.7)	13 208 (5.3)	1906 (11.0)	<0.0001
Dementia	21 792 (8.2)	19 481 (7.9)	2311 (13.3)	<0.0001
Chronic pain	63 231 (23.9)	57 582 (23.3)	5649 (32.6)	<0.0001
Cancer	83 941 (31.7)	77 425 (31.3)	6516 (37.6)	<0.0001
Alcohol abuse	5199 (2.0)	4452 (1.8)	747 (4.3)	<0.0001
Substance abuse	2155 (0.8)	1754 (0.7)	401 (2.3)	<0.0001
Medication				
0–4	78 401 (29.6)	75 479 (30.5)	2922 (16.9)	<0.0001
5–9	112 106 (42.4)	106 173 (43.0)	5933 (34.2)	–
≥10	73 966 (28.0)	65 489 (26.5)	8477 (48.9)	–
Benzodiazepine	65 362 (24.7)	59 260 (24.0)	6102 (35.2)	<0.0001
Antipsychotic	15 758 (6.0)	13 991 (5.7)	1767 (10.2)	<0.0001
Opioid	18 336 (6.9)	16 268 (6.2)	2068 (11.3)	<0.0001
Past ED visits				
0	84 638 (32.0)	83 103 (33.6)	1535 (8.9)	<0.0001
1–2	107 385 (40.6)	102 999 (41.7)	4386 (25.3)	–
3–4	41 787 (15.8)	37 962 (15.4)	3825 (22.1)	–
5–9	25 745 (9.7)	20 536 (8.3)	5209 (30.1)	–
≥10	4918 (1.7)	2541 (1.0)	2377 (13.7)	–
Past hospitalizations				
0–1	195 675 (74.0)	186 421 (75.4)	9254 (53.4)	<0.0001
≥2	68 798 (26.0)	60 720 (24.6)	8078 (46.6)	–
Hospitalization length of stay				
None	132 859 (51.0)	127 829 (52.5)	5030 (29.9)	<0.0001
1–2	52 088 (20.0)	49 049 (20.1)	3039 (18.1)	–
≥3	75 555 (29.0)	66 788 (27.4)	8767 (52.1)	–
General practitioner	191 583 (72.4)	179 266 (72.5)	12 317 (71.1)	<0.0001
Past visits to general practitioner				
0–4	65 579 (24.8)	61 894 (25.0)	3685 (21.3)	<0.0001
5–9	109 186 (41.3)	103 042 (41.7)	6144 (35.5)	–
10–14	53 252 (20.1)	49 456 (20.0)	3796 (21.9)	–
≥15	36 456 (13.8)	32 749 (13.3)	3707 (21.4)	–
Past visits to specialists				
0–4	113 391 (42.9)	108 864 (44.1)	4527 (26.1)	<0.0001
5–9	67 448 (25.5)	63 134 (25.6)	4314 (24.9)	–
10–14	36 107 (13.7)	75 143 (30.4)	8491 (49.0)	–
≥15	47 527 (18.0)	41 993 (17.0)	5534 (31.9)	–
Residential area				
Metropolitan area	165 960 (63.0)	156 043 (63.3)	9917 (57.5)	<0.0001
Small town	38 011 (14.4)	35 482 (14.4)	2529 (14.7)	–
Rural	59 686 (22.6)	54 879 (22.3)	4807 (27.9)	–
Material deprivation				
Q1	39 968 (15.1)	37 910 (15.3)	2058 (11.9)	<0.0001
Q2	46 122 (17.4)	43 360 (17.5)	2762 (15.9)	–
Q3	48 880 (18.5)	45 787 (18.5)	3093 (17.9)	–
Q4	55 817 (21.1)	52 033 (21.1)	3784 (21.8)	–
Q5	60 220 (22.8)	55 594 (22.5)	4626 (26.7)	–
Social deprivation				
Q1	40 194 (15.2)	37 767 (15.3)	2427 (14.0)	<0.0001
Q2	43 031 (16.3)	40 423 (16.4)	2608 (15.1)	–
Q3	50 122 (19.0)	46 942 (19.0)	3180 (18.4)	–
Q4	54 994 (20.8)	51 427 (20.8)	3567 (20.6)	–
Q5	62 666 (23.7)	58 125 (23.5)	4541 (26.2)	–

Percentages in parenthesis are relative to the column total.

† Groups were compared using the χ^2^‐test.

‡ Groups were compared using the Kruskal–Wallis test.

ACSC, ambulatory care sensitive condition; COPD, chronic obstructive pulmonary disease; ED, emergency department.

Table 3 presents the results from the logistic regression model. The variables significantly associated with frequent ED use among older adults are older age, presence of COPD or diabetes, higher comorbidity index, common mental‐health disorders, polypharmacy and severe polypharmacy, higher number of past specialist visits, a rural residence, and higher material and social deprivation, with greater number of past ED visits being the most strongly associated (OR of 7.02 for 5–9 visits and OR of 20.83 for ≥10 visits). Furthermore, dementia and hospitalization length of stay are negatively associated with frequent ED use.

**Table 3:** Multivariate logistic regression results: factors associated with frequent ED use among older adults with ambulatory care sensitive conditions

Variables	OR	CI (99%)
Age	1.02	(1.01–1.02)†
Sex		
Female	0.96	(0.91–1.01)
ACSC Coronary heart disease	1.04	(0.98–1.01)
ACSC Congestive heart failure	1.08	(0.99–1.17)
ACSC COPD	1.27	(1.19–1.36)†
ACSC Asthma	1.05	(0.95–1.16)
ACSC Diabetes	1.12	(1.05–1.18)†
ACSC High blood pressure	0.94	(0.88–0.99)†
ACSC Epilepsy	1.06	(0.88–1.27)
Comorbidity index		
0	Reference	
1–2	1.19	(1.10–1.28)†
3–4	1.31	(1.20–1.44)†
≥ 5	1.37	(1.24–1.51)†
Common mental–health disorder	1.09	(1.02–1.17)†
Severe mental disorder	0.98	(0.89–1.08)
Dementia	0.87	(0.80–0.96)†
Chronic pain	1.05	(0.99–1.11)
Cancer	0.99	(0.93–1.04)
Alcohol abuse	1.07	(0.92–1.24)
Substance abuse	1.19	(0.96–1.48)
Medication		
0–4	Reference	
5–9	1.13	(1.05–1.21)†
≥10	1.47	(1.35–1.60)†
Benzodiazepine	1.08	(1.01–1.14)†
Antipsychotic	1.01	(0.91–1.12)
Opioid	1.09	(1.00–1.20)
Past ED visits		
0	Reference	
1–2	1.86	(1.71–2.02)†
3–4	3.51	(3.20–3.85)†
5–9	7.14	(6.48–7.87)†
≥10	21.02	(18.14–24.37)†
Hospitalization total length of stay		
None	Reference	
1–2 days	0.95	(0.88–1.03)
≥3 days	0.90	(0.83 0.97)†
General practitioner	0.99	(0.94–1.05)
Past visits to general practitioner		
0–6	Reference	
≥7	1.01	(0.96–1.07)
Past visits to specialists		
0–6	Reference	
≥7	1.16	(1.09–1.23)†
Residential area		
Metropolitan area	Reference	
Small town	1.04	(0.97–1.12)
Rural	1.19	(1.11–1.28)†
Material deprivation		
Q1	Reference	
Q2	1.06	(0.96–1.16)
Q3	1.08	(0.97–1.19)
Q4	1.15	(1.05–1.26)†
Q5	1.19	(1.08–1.30)†
Social deprivation		
Q1	Reference	
Q2	0.99	(0.90–1.09)
Q3	1.05	(0.96–1.15)
Q4	1.08	(0.99–1.18)
Q5	1.13	(1.03–1.23)†

† Statistical significance ACSC, ambulatory care sensitive condition; CI, confidence interval; COPD, chronic obstructive pulmonary disease; OR, odds ratio.

## Discussion

The findings of the present study yield a comprehensive portrait of frequent geriatric ED users, and addresses several gaps reported in the literature. We found that the variables mainly associated with frequent geriatric ED use are higher number of specialist visits, higher comorbidity index, severe polypharmacy (≥10 medications) and higher number of past ED visits. Dementia was inversely associated with frequent ED use.

Many of the present results support past studies. First, prior ED visits are associated with frequent ED use, past use of healthcare being one of the most important variables influencing healthcare use.^6^, ^11^ In addition, a recent study by Castillo *et al*. showed that a higher comorbidity score was associated with frequent geriatric ED use.^12^ Considered a proxy for comorbidity, a higher number of prescribed drugs was also an important variable.^6^ Finally, living in a rural area (where service availability might differ from urban areas), and higher social and material deprivation (associated with higher unmet healthcare needs) were also pointed out as associated variables^6^, ^11^

A systematic literature review by Giannouchos *et al*., which aimed at identifying the characteristics of frequent adult ED users, presents comparable results.^13^ Indeed, some variables were associated with both frequent adult and geriatric ED use: high past ED use, higher comorbidity index, higher prevalence of COPD, low socioeconomic situation and the presence of common mental health disorders. The number of primary care provider visits, the presence of severe mental health disorders, and alcohol and substance abuse were, however, associated with frequent ED use among the adult population, but not in the present results (older people).^13^ Important differences between these populations should, however, be noted. The geriatric population accounts for approximately 16% of the Canadian population, but represents up to 40% of ED gurney patients (compared with 26% for the adult population). The hospital admission rate after ED visits is also higher in the geriatric population (45%). Indeed, the geriatric population is more prone to urgent and semi‐urgent ED visits requiring specialized care and to present adverse effects related to their ED visits.^14^ In addition, the lower hospital admission rate for frequent adult ED users^13^ suggests there might be higher rates of avoidable ED visits and unmet care needs in primary‐care services.^15^ Despite similarities, the reported differences show the relevance of developing interventions that take into account each of the population's characteristics and specific healthcare needs.

Some of the present results represent specific geriatric challenges. First, having dementia is negatively associated with frequent ED use in our results. Empirical evidence regarding this result are mixed in the literature. To begin with, an integrative review by Hunt *et al*. stated that patients with dementia had higher rates of ED visits and hospitalization.^16^ Their high level of multimorbidity compared with the cognitively intact population helped explain their healthcare use.^17^, ^18^ Higher odds of potentially avoidable ED visits were also reported in this population.^16^, ^19^ In contrast, an American study by Morris *et al*. also reported Alzheimer's disease as a protective factor of ED use among older adults.^20^ It could be posited that a dementia diagnosis implies complex clinical challenges, so that it would not come out as a protective factor of frequent ED use on its own. If increased dementia symptoms and the associated disabilities play a role in increased ED visits,^18^ the effective management of needs related to dementia might decrease ED use.^16^ Indeed, older adults with dementia tend to benefit from home care follow up – a way to prevent avoidable ED visits – by including adequate evaluation of both physical and cognitive needs.^21^ Some variables, such as a higher level of care coordination and specialty dementia care, were also associated with lower ED use.^16^ The absence of variables, such as home care service, prevents us from drawing any further conclusions.

Furthermore, variables related to primary healthcare (e.g. having a GP and the number of visits to a GP) were not shown to be significant. Palmer *et al*. showed that frequent ED users were more likely to have a primary care provider, whereas Sandoval *et al*. stated that having a GP was not related to ED use.^22^, ^23^ Timely and effective follow up in primary healthcare services for ACSC has been associated with a decrease in potentially preventable ED visits and hospitalizations. Increased ambulatory visits and continuity of care were also associated with lower rates of ED visits among geriatric patients, as a patient closely managed by primary care providers might tend to seek care from them.^24^ We can also hypothesize that a large proportion of ED visits by older adults in our population could be attributed to acute problems that could not have been prevented through follow up in primary healthcare services.^25^, ^26^ As stated by Street *et al*., this might suggest that frequent users with higher comorbidity indices are at greater risk of exacerbations and complications, requiring rapid access to advanced care. Furthermore, higher comorbidity was also associated with more specialist referrals and visits in the geriatric population.^27^ Patients with comorbidities generally have more consultations with specialists than with primary care providers, as they require specialized care.^28^ We did not, however, report the type of specialist consulted or patient diagnoses.

Medico‐administrative databases provide access to a range of variables evaluated by professionals and the opportunity to generalize the results to large populations. Nevertheless, they do not give a comprehensive picture of the characteristics of our population, as they do not provide access to self‐perceived variables. The latter provide a measurement of the overall health status of individuals, which would better predict health‐seeking and health service behaviors than clinical measures.^29^ In this sense, the combination of medico‐administrative data and questionnaires administered to the population (e.g. national survey data) would optimize the understanding, description and prediction of clinical outcomes. Pairing data from multiple sources is of growing interest for policymakers, clinicians and researchers, as it could support accurate measurement of clinical performance and patient health results, as well as help design interventions based on relevant variables.^29^

Frequent geriatric users, as frequent users in general, are a heterogeneous and complex population. Undertaking effective intervention requires adequate knowledge of this population and its characteristics, and is paramount to achieving better health outcomes and reducing the risk of adverse outcomes. The present findings provide an opportunity to improve the care of geriatric patients who frequently use acute care services by highlighting specific variables related to their use of ED services. To control costs and ensure the highest quality of care, we must meet the needs of geriatric patients in the ED and also across the care continuum. As an example, adequate case management interventions could lead to fewer ED visits by frequent users by improving ACSC management, as well as through the orientation and coordination of healthcare services.^30^

The present study has some notable strengths. First, it was carried out with an exhaustive medico‐administrative database of older adults in Quebec, Canada, making the results generalizable to our whole population of interest. It also addresses a gap in the literature by selecting a set of independent variables, some of which have received little attention. The use of a medico‐administrative database also has inherent limitations: the lack of some variables, including self‐perceived variables, specifically perception of quality of life and disease severity. Nevertheless, we overcame the absence of socioeconomic variables by using a proxy for material and social deprivation. In addition, we examined frequent ED use as an outcome, without regard for the appropriateness of the ED visits. It could have given a better idea of to what an extent a primary care visit could contribute to preventing frequent ED use.

Frequent geriatric ED users are a distinct and complex population whose characteristics need to be understood and thoroughly managed in order to enhance the quality and efficiency of the care they receive. Despite helping to produce an accurate and generalizable portrait of this population, the use of administrative databases has limitations, such as the type of available variables. Further studies should address the characterization of this population, especially with databases combining self‐perceived and professionally evaluated variables.

## Disclosure statement

The authors declare no conflict of interest.
